# Sex-specific influences shaping the development and trajectory of psychosis

**DOI:** 10.1192/j.eurpsy.2026.10321

**Published:** 2026-07-31

**Authors:** L.-K. Pries

**Affiliations:** Department of Psychiatry and Psychotherapy, Faculty of Medicine and University Hospital of Cologne, Cologne, Germany

## Abstract

**Abstract:**

Early life experiences, particularly childhood adversity, can have enduring effects on mental health. Males and females differ in their vulnerability to specific adversities, symptom expression, and the mechanisms linking adversity to psychopathology. For example, certain symptoms may be more strongly associated with abuse in females and with neglect in males, reflecting sex-specific patterns of risk. Moreover, the developmental pathways through which adversity contributes to psychopathology may also differ by sex. In females, one pathway linking early adversity to later psychosis-related outcomes may involve pubertal timing, particularly age at menarche. Puberty represents a critical developmental period during which environmental exposures interact with biological maturation, shaping hormonal, neural, and psychosocial processes. As such, pubertal timing may function as a sex-specific mediator of risk, helping to explain why females exposed to early adversity show heightened susceptibility to certain symptoms. Previous research suggests that childhood adversity is associated with earlier menarche, which in turn increases vulnerability to both internalizing and externalizing difficulties. However, the role of pubertal timing in the development of psychosis-related outcomes remains underexplored. This presentation reports new findings on sex differences in the impact of environmental exposures on symptom profiles, as well as female-specific developmental mechanisms shaping mental health outcomes. By elucidating how early adversity interacts with pubertal timing and other sex-specific pathways, this work contributes to improved early identification, prevention, and targeted intervention strategies.

**Disclosure of Interest:**

None Declared

